# Supplementary material for: Between-species variation in neocortical sulcal anatomy of the carnivoran brain
Source: eLife. 2026 Jan 13;13:RP100851. doi: 10.7554/eLife.100851 (PMC12799212; doi:10.7554/eLife.100851)
Supplement: Figure 5—source data 1. [file elife-100851-fig5-data1.docx]

| **Figure 5 - source data 1**. Effects of forepaw dexterity and sociality on relative length of the proreal sulcus | | | | | |
| --- | --- | --- | --- | --- | --- |
| Predictor | Reference sulcus | Hemisphere | *F (df_num_, df_den_)* | *p* | η²ₚ |
| Forepaw dexterity | ansate | right | 1.23 (1, 22) | .28030 | .13 |
| Sociality | ansate | right | 25.43 (1, 22) | **< .00001** | .54 |
| Forepaw dexterity | coronal | right | 1.57 (1, 22) | .22380 | .16 |
| Sociality | coronal | right | 32.53 (1, 22) | **< .00001** | .60 |
| Forepaw dexterity | marginal | right | 1.39 (1, 22) | .25050 | .15 |
| Sociality | marginal | right | 28.91 (1, 22) | **< .00001** | .57 |
| Forepaw dexterity | presylvian | right | 1.75 (1, 22) | .19890 | .18 |
| Sociality | presylvian | right | 36.42 (1, 22) | **< .00001** | .62 |
| Forepaw dexterity | retrosplenial | right | 1.61 (1, 22) | .21740 | .17 |
| Sociality | retrosplenial | right | 33.47 (1, 22) | **< .00001** | .60 |
| Forepaw dexterity | splenial | right | 1.72 (1, 22) | .20370 | .18 |
| Sociality | splenial | right | 35.62 (1, 22) | **< .00001** | .62 |
| Forepaw dexterity | suprasylvian | right | 1.53 (1, 22) | .22880 | .16 |
| Sociality | suprasylvian | right | 31.81 (1, 22) | **< .00001** | .59 |
| Forepaw dexterity | ansate | left | .78 (1, 22) | .38580 | .09 |
| Sociality | ansate | left | 16.25 (1, 22) | **.00060** | .42 |
| Forepaw dexterity | coronal | left | .87 (1, 22) | .36220 | .10 |
| Sociality | coronal | left | 17.97 (1, 22) | **.00030** | .45 |
| Forepaw dexterity | marginal | left | .88 (1, 22) | .35900 | .10 |
| Sociality | marginal | left | 18.22 (1, 22) | **.00030** | .45 |
| Forepaw dexterity | presylvian | left | .92 (1, 22) | .34820 | .10 |
| Sociality | presylvian | left | 19.07 (1, 22) | **.00020** | .46 |
| Forepaw dexterity | retrosplenial | left | .58 (1, 22) | .45290 | .07 |
| Sociality | retrosplenial | left | 12.12 (1, 22) | **.00210** | .36 |
| Forepaw dexterity | splenial | left | .93 (1, 22) | .34610 | .10 |
| Sociality | splenial | left | 19.24 (1, 22) | **.00020** | .47 |
| Forepaw dexterity | suprasylvian | left | .91 (1, 22) | .35070 | .10 |
| Sociality | suprasylvian | left | 18.87 (1, 22) | **.00030** | .46 |
| *Note*. Linear models were used to test the effects of two categorical behavioural predictors: forepaw dexterity (low, high) and sociality (solitary, cooperative hunting) on the relative length of the proreal sulcus. Models were fit separately for each hemisphere (left, right) and each target-reference sulcus pair. Significant *p*-values (bolded) indicate greater relative sulcal length in species with high dexterity or cooperative hunting behaviour. Proportions of significant results are presented in **Figure 5 - figure supplement 1**. P *df*_num_, degrees of freedom numerator; *df*_den_ degrees of freedom denominator; η²ₚ, partial eta squared | | | | | |
