# Supplementary material for: Between-species variation in neocortical sulcal anatomy of the carnivoran brain
Source: eLife. 2026 Jan 13;13:RP100851. doi: 10.7554/eLife.100851 (PMC12799212; doi:10.7554/eLife.100851)
Supplement: Figure 5—source data 2. [file elife-100851-fig5-data2.docx]

| **Figure 5 - source data 2**. Effects of forepaw dexterity and sociality on relative length of the postcruciate sulcus | | | | | |
| --- | --- | --- | --- | --- | --- |
| Predictor | Reference sulcus | Hemisphere | *F (df_num_, df_den_)* | *p* | η²ₚ |
| Forepaw dexterity | marginal | right | 71.71 (1, 22) | **< .00001** | .75 |
| Sociality | marginal | right | 5.28 (1, 22) | **.03150** | .19 |
| Forepaw dexterity | retrosplenial | right | 46.79 (1, 22) | **< .00001** | .66 |
| Sociality | retrosplenial | right | 3.94 (1, 22) | .05970 | .15 |
| Forepaw dexterity | splenial | right | 56.22 (1, 22) | **< .00001** | .70 |
| Sociality | splenial | right | 4.09 (1, 22) | .05560 | .16 |
| Forepaw dexterity | suprasylvian | right | 52.06 (1, 22) | **< .00001** | .69 |
| Sociality | suprasylvian | right | 4.72 (1, 22) | **.04080** | .18 |
| Forepaw dexterity | marginal | left | 38.24 (1, 22) | **< .00001** | .64 |
| Sociality | marginal | left | .00 (1, 22) | .97190 | .00 |
| Forepaw dexterity | retrosplenial | left | 30.57 (1, 22) | **< .00001** | .59 |
| Sociality | retrosplenial | left | .00 (1, 22) | .96130 | .00 |
| Forepaw dexterity | splenial | left | 84.59 (1, 22) | **< .00001** | .79 |
| Sociality | splenial | left | .61 (1, 22) | .44400 | .03 |
| Forepaw dexterity | suprasylvian | left | 96.33 (1, 22) | **< .00001** | .82 |
| Sociality | suprasylvian | left | .04 (1, 22) | .84340 | .00 |
| *Note*. Linear models were used to test the effects of two categorical behavioural predictors: forepaw dexterity (low, high) and sociality (solitary, cooperative hunting) on the relative length of the postcruciate sulcus. Models were fit separately for each hemisphere (left, right) and each target-reference sulcus pair. Significant *p*-values (bolded) indicate greater relative sulcal length in species with high dexterity or cooperative hunting behaviour. Proportions of significant results are presented in **Figure 5 - figure supplement 1**. P *df*_num_, degrees of freedom numerator; *df*_den_ degrees of freedom denominator; η²ₚ, partial eta squared | | | | | |
