# Supplementary material for: Between-species variation in neocortical sulcal anatomy of the carnivoran brain
Source: eLife. 2026 Jan 13;13:RP100851. doi: 10.7554/eLife.100851 (PMC12799212; doi:10.7554/eLife.100851)
Supplement: Figure 5—source data 3. [file elife-100851-fig5-data3.docx]

| **Figure 5 - source data 3**. Effects of forepaw dexterity and sociality on relative length of the cruciate sulcus | | | | | |
| --- | --- | --- | --- | --- | --- |
| Predictor | Reference sulcus | Hemisphere | *F (df_num_, df_den_)* | *p* | η²ₚ |
| Forepaw dexterity | marginal | right | 7.24 (1, 22) | **.01330** | .25 |
| Sociality | marginal | right | .00 (1, 22) | .96900 | .00 |
| Forepaw dexterity | retrosplenial | right | 1.45 (1, 22) | .24210 | .09 |
| Sociality | retrosplenial | right | 2.36 (1, 22) | .13870 | .10 |
| Forepaw dexterity | splenial | right | 5.21 (1, 22) | **.03240** | .22 |
| Sociality | splenial | right | 1.31 (1, 22) | .26500 | .06 |
| Forepaw dexterity | suprasylvian | right | 7.63 (1, 22) | **.01130** | .28 |
| Sociality | suprasylvian | right | 1.09 (1, 22) | .30680 | .05 |
| Forepaw dexterity | marginal | left | 12.15 (1, 22) | **.00210** | .37 |
| Sociality | marginal | left | .12 (1, 22) | .72980 | .01 |
| Forepaw dexterity | retrosplenial | left | .80 (1, 22) | .38200 | .05 |
| Sociality | retrosplenial | left | 2.11 (1, 22) | .16080 | .09 |
| Forepaw dexterity | splenial | left | 8.19 (1, 22) | **.00910** | .29 |
| Sociality | splenial | left | .32 (1, 22) | .57990 | .01 |
| Forepaw dexterity | suprasylvian | left | 19.41 (1, 22) | **.00020** | .50 |
| Sociality | suprasylvian | left | 3.06 (1, 22) | .09390 | .12 |
| *Note*. Linear models were used to test the effects of two categorical behavioural predictors: forepaw dexterity (low, high) and sociality (solitary, cooperative hunting) on the relative length of the cruciate sulcus. Models were fit separately for each hemisphere (left, right) and each target-reference sulcus pair. Significant *p*-values (bolded) indicate greater relative sulcal length in species with high dexterity or cooperative hunting behaviour. Proportions of significant results are presented in **Figure 5 - figure supplement 1**. P *df*_num_, degrees of freedom numerator; *df*_den_ degrees of freedom denominator; η²ₚ, partial eta squared. | | | | | |
